# Progress in X-Ray Therapeutics

**Published:** 1902-09-06

**Authors:** 


### Progress in X-Ray Therapeutics.

(*Concluded from page* 380.)

**Sarcoma.**—W. J. Morton[5] records the case of a girl who had had her left arm amputated for sarcoma of the elbow joint (round-celled sarcoma microscopically), and who came to him with her right elbow joint affected in a precisely similar manner, a radiogram of the joint having exactly the same appearance as a radiogram of the amputated joint prior to operation. After two months' treatment the elbow presented no symptoms of trouble whatever, and there was normal use of the arm. Another case he reports is one of sarcoma of the side of the head, in which there was a hard bony tumour in front of the ear, and a soft fungating tumour behind the ear. After a month's treatment the hard bony tumour was distinctly more conical in shape due to shrinkage round its base, and merely a a small lump of the soft tumour remained. In this case the relief of pain was a most marked symptom, for whereas at the beginning of treatment the patient was in the habit of taking half a grain of morphia every two or three hours, at the end of a month "morphia taken, when at all, not for pain but from long habit." W. B. Coley [3] mentions several cases, in three of which the X-rays had no effect whatever, but in one of lympho-sarcoma which had resisted the toxin treatment, after three weeks' treatment with the X-rays, the improvement was "little short of marvellous;" and in another, where a man had been operated on five times for round-cell sarcoma, the improvement that took place was "most remarkable."

**Epithelioma.**—C. W. Allen, in a paper on "Cutaneous Epithelioma,"[8] gives several cases greatly improved or cured. In five cases, four recurrent and one primary, all might be considered cured. In another case of extensive recurrence the growth decreased nearly one-half in size, and in another case of many years' standing, the disease appeared to be cured. He also quotes cases either cured or relieved given by other authors. C. R. C. Lyster [4] also gives a case of epithelioma of the lip which was apparently cured.

**Rodent Ulcer.**—There seems to be a universal consensus of opinion of the value of the X-ray in this disease: thus G. H. Lancashire [5] says, "if it were only for the results achieved in dealing with this distressing disease, the X-ray treatment deserves special recognition." The only thing yet undecided in his opinion is, are the cures permanent? He quotes two cases of four and six months respectively, in which the patients had remained free from the disease after being discharged from treatment. Malcolm Morris [5] gives a case of a small rodent ulcer of the cheek, which disappeared after seven applications of the Finsen light, but which recurred after an interval of about a year. It was then treated with the X-rays, and after ten applications the crust over the sore was easily removed, and "revealed a smooth red surface with no elevation or depression of the epidermis, and no underlying or surrounding induration." He gives a second case which recurred a year and a half after an apparent cure with the Finsen light. This author says "even very extensive ulcerations of the rodent type rapidly heal by granulation under treatment by X-rays, and there can be no doubt as to the advantage of employing this mode of treatment." J. W. Pugh [6] records four cases of apparently perfect cure, two of them being in very old men, one of them being 93 years of age, and the other 83.

**Lupus.**—Most authors seem to consider the X-ray as a secondary mode of treatment to the arc light in this disease. Thus Malcolm Morris and S. E. Dore [9] say "although we have found the X-rays very useful as an adjunct to the light rays, we do not, except in certain cases, regard them as an adequate substitute. We have treated many cases with the X-rays alone, and have formed the conclusion that in the majority of cases Finsen's method is more reliable, and apparently gives more permanent results." They say, however, that the rays are effective in ulcerating cases, as "the ulcerations heal rapidly." Also, for mucous membranes inaccessible to the light, they are very useful. G. H. Lancashire [9] thinks that "most cases of ulcerated lupus call for X-ray treatment," and that in widespread and extensive cases the treatment is more especially indicated. G. H. Rodman [10] gives a most successful case of cure of a long-standing and severe case of lupus. The treatment was begun April 19th, 1901, and the patient was completely cured on July 29th, 1901, and she remained quite well in the following September. T. C. Squance [10] also records a successful case in a girl of 17.

**Callous Sinuses.**—Berry Hart [5] relates two most suggestive cases of the rapid cure of callous sinuses of the abdominal wall persisting after operation by the application of the X-rays. He discovered this action of the rays by accident, in a case, in which in order to find out if possible the direction of a sinus in the right iliac fossa, he had a skiagram taken with a probe inserted in the sinus. After this he was struck with the rapidity with which the sinus healed without any further treatment. After treating a second case in which there was a sinus which had been present for some months with the X-rays, he got an equally satisfactory result, the sinus closing after three applications of the rays. The writer has had two cases which have healed rapidly under this method of treatment; one, a sinus of two months' standing after an operation for appendicitis, which healed up after three applications of the rays, and one a small ulcer, which had persisted for some months in the scar in the abdominal wall after a laparotomy for tubercular peritonitis.

[5] Brit. Med. Journ., May 31, 1902. [6] Brit. Med. Journ., April 12, 1902. [7] Brit. Med. Journ., Feb. 8, 1902. [8] Med. Rec., Jan. 25, 1902. [9] Brit. Med. Journ., May 31, 1902. [10] Lancet, Nov. 16, 1901.

